# Change and Stability in Sibling Resemblance in Obesity Markers: The Portuguese Sibling Study on Growth, Fitness, Lifestyle, and Health

**DOI:** 10.1155/2019/2432131

**Published:** 2019-11-20

**Authors:** Sara Pereira, Peter T. Katzmarzyk, Donald Hedeker, José Maia

**Affiliations:** ^1^CIFI^2^D, Faculty of Sport, University of Porto, Porto 4200-450, Portugal; ^2^Pennington Biomedical Research Center, Louisiana State University, Baton Rouge, LA 70808, USA; ^3^Department of Public Health Sciences, University of Chicago, Chicago, IL 60637, USA

## Abstract

**Background/Objectives:**

Obesity markers evolve over time and these changes are shared within the family orbit and governed by individual and environmental characteristics. Available reports often lack an integrated approach, in contrast to a multilevel framework that considers their concurrent influence. Hence, this study aims to (1) describe mean changes in obesity markers (body fat (%BF), body mass index (BMI), and waist circumference (WC)) across sib-ships; (2) analyze tracking of individuals within their sib-ship in these markers during 2 years of follow-up; (3) probe consistency in sibling resemblance in these markers; and (4) analyze the joint influence of individual and familial characteristics in these markers.

**Subjects/Methods:**

The sample comprises 168 biological Portuguese siblings (brother-brother (BB), sister-sister (SS), and brother-sister (BS)) aged 9–17 years. %BF, BMI, and WC were measured using standardized protocols, and biological maturation was assessed. Physical activity, diet, screen time, and familial characteristics were obtained by questionnaires. Multilevel models were used to analyze the clustered longitudinal data. Sibling resemblance was estimated with the intraclass correlation.

**Results:**

On average, all sib types increased in BMI and WC over 2 years of follow-up, and SS pairs increased in %BF. Individuals within sib-ships track high in all obesity markers across time. Consistency in siblings' resemblance was also noted, except for BB pairs in %BF which decreased at follow-up. More maturing siblings tend to have higher values in all markers. Greater screen time was associated with higher %BF, whereas those consuming more sugary drinks had lower %BF and BMI values. Siblings whose mothers had less qualified occupations tended to have lower BMI values.

**Conclusions:**

Longitudinal individual tracking and sibling resemblance for obesity markers were found. Yet, different trajectories were also identified depending on the marker and sib type. Individual and familial characteristics exert different influences on each obesity marker.

## 1. Introduction

There is a strong call to investigate how changes in obesity markers such as percent body fat (%BF), body mass index (BMI), and waist circumference (WC) occur during childhood and adolescence to better comprehend their etiology and tendencies and to identify important time windows for fruitful interventions [1]. Regarding the development of obesity, three critical periods can be identified, namely, prenatal period, infancy, and adolescence [2]. Further, it is well known that adolescence is a period of rapid and systematic changes in body size, shape, and composition, where BF distribution and patterns vary considerably with age, sex, and biological maturation [3]. The etiology of obesity is multifactorial and complex, stemming from the additive and interactive links between the individual (biological and behavioral) and its environmental (familial, social, and geographic) characteristics [4].

Family members share a multitude of traits which make them highly suitable candidates to tease out the influences of genes and the environment on obesity markers [5]. For example, twin data showed that BMI heritability estimates range from 70 to 90% in both adolescent boys and girls across the globe [6]. However, other studies using complex twin models showed inconsistent results regarding the additive components of genetic, unique, and shared environmental factors for BMI and WC across different populations [7–9]. Furthermore, intraclass correlation coefficients (ICCs) from twin studies consistently show higher monozygotic twins' resemblance than in dizygotic twins of both sexes for BMI [9–11], WC [9, 10], and %BF [8]. Additionally, data from nuclear families revealed that ICC values depend on the kinship structure [12–15] and may vary from 0.15 (spouses) to 0.44 (father-daughter) in BMI, 0.11 (father-daughter) to 0.53 (siblings) in WC, and 0.05 (spouses) to 0.34 (both father-daughter and mother-daughter) in %BF.

Apparently, there is a paucity of studies related to changes in obesity markers grounded on twins and nuclear family data. Further, available results show discrepancy in the sizes of both genetic and environmental effects [11, 16, 17]. For example, Lajunen et al. [11] using a Finish twin cohort reported that the amount of BMI variation explained by additive genetic factors changed from 0.69 to 0.83 in boys and 0.58 to 0.74 in girls. Moreover, shared environment decreased from 0.15 to 0.00 in boys and 0.21 to 0.03 in girls, whereas unique environment tended to have similar values across time, varying from 0.15 to 0.17 in boys and 0.21 to 0.23 in girls aged 11–17 years. However, Haberstick et al. [16] using a combined sample of twins and siblings showed that the explained variance by the genetic effects slightly decreased in boys from 0.96 to 0.89, whereas in girls it remained stable across time (0.97). Moreover, the effect of the unique environment decreased in both sexes (0.51 to 0.29 and 0.66 to 0.33 in boys and girls, respectively). Additionally, Hunt et al. [17] using familial data from the Canada Fitness Survey reported a significant level of resemblance among families based on heritability estimates for body mass, BMI, skinfolds, and WC. Although the intraclass correlation coefficients between family members were in general low at baseline, they also remained lower with the 7-year change with slight variations depending on the kinship structure and obesity markers.

We contend that a fruitful approach to investigate changes and/or stability in obesity markers should be simultaneously grounded on longitudinal data, with related individuals, using individual-based and environmental-based characteristics as covariates. For this, the multilevel model approach to investigate siblings' obesity markers in a 2-year follow-up period and their associations with biological and familial covariates is well suited to provide novel and relevant information that can be used for planning and developing more effective intervention programs within the family orbit. Therefore, we aim (1) to describe mean changes in obesity markers (%BF, BMI, and WC) across sib-ships; (2) to analyze tracking of individuals within their sib-ship in obesity markers during 2 years of follow-up; (3) to investigate consistency in sibling resemblance in their obesity markers; and (4) to analyze the joint influence of individual and familial characteristics in these markers.

## 2. Methods

### 2.1. Study Design and Participants

The study sample comprised young siblings (aged 9–17 years) from the Portuguese Sibling Study on Growth, Fitness, Lifestyle, and Health. In brief, this study investigates physical growth, body composition, physical fitness, physical activity, metabolic syndrome, and health behaviors in a cohort of siblings based on cross-sectional and longitudinal information [18]. All participants enrolled in this study were part of a larger project named The Portuguese Healthy Family Study [19]. For the present article, we used available longitudinal data on 474 siblings followed up for three consecutive years (2011–2013). However, the final sample was only composed by 168 siblings (74 females and 94 males) from 84 families (58 siblings and 26 twins) which had complete data at baseline (2011) and follow-up (2013) for biological, behavioral, and familial characteristics. No statistically significant mean differences (*p* > 0.05) were observed between included and excluded siblings in height, weight, and percentage of body fat (%BF), body mass index (BMI), and waist circumference (WC).

The assessment of twins' zygosity usually requires collecting biological samples (e.g., blood or cheek swabs), but it can be fairly assessed with reliable questionnaires. Yet, due to limited time as well as other operational constraints inherent to each school setting during data collection, we were not able to send a putative cross-culturally validated questionnaire to twins' mothers, aiming to classify their zygosity.

All recruitment and data collection were approved by the Ethics Committee of the University of Porto and school authorities and the written informed consent was acquired from legal guardians for all participants.

## 3. Results

### 3.1. Measures

#### 3.1.1. Anthropometry and Body Composition

Height, weight, and WC were measured using standardized protocols established by the International Society for the Advancement of Kinanthropometry [20]. %BF was estimated using a reliable and valid instrument [21]—a portable bioelectrical impedance scale (TANITA BC- 418 MA Segmental Body Composition Analyzer Tanita Corporation, Japan). All youth were barefoot and in light clothing. BMI was computed using the standard formula: BMI = weight (kg)/height (m).^2^

### 3.2. Individual Characteristics

#### 3.2.1. Biological Maturation

Maturity offset was used to assess biological maturation. This procedure is valid and reliable [22] and has been commonly used in children and youth [23]. Briefly, the maturity offset estimates the temporal distance (in decimal years) from age-at-peak height velocity (PHV). A positive (+) maturity offset indicates the number of years the participant is beyond PHV, whereas a negative (−) maturity offset represents the number of years the participant is before PHV.

#### 3.2.2. Diet

Diet consumption was obtained using a food frequency questionnaire (FFQ) adapted and modified from the Health Behavior in School-aged Children Survey (HBSC) [24] using typical Portuguese food items. This questionnaire has been broadly applied in multicountry studies [25]. Youth were asked about various types of food consumed in a typical week. Food items related to healthy diet are fruits, vegetables, dark-green vegetables, orange vegetables, fruit juice, skimmed milk, low-fat milk, whole milk, cheese, other milk products, bread or whole grains, beans, lentils, bean curd, eggs, and fish. Food items related to unhealthy diet comprehend sweets, sugary drinks, cakes, pastries, donuts, diet sodas, ice cream, potato chips, French fries, fast foods sports drinks, energy drinks, and fried food. For each item, the reported answers were converted into weekly portions as follows: “never” = 0; “less than once per week” = 0.5; “once per week” = 1; “2–4 days per week” = 3; “5–6 days per week” = 5,5; “once a day, every day” = 7; and “more than once a day” = 10, as previously advocated [26].

### 3.3. Physical Activity

Total physical activity (TPA) was assessed with the Baecke questionnaire [27]. This is a reliable and valid instrument [28] and includes three specific domains (work/school PA, leisure-time PA, and sports participation) which are based on a total of 16 Likert-type questions. TPA was estimated based on the sum of these three specific domains. For each domain, each score ranges from 1 (minimal) to 5 (maximal), such that the TPA score varies between 3 and 15. Participants answered the questionnaire during regular physical education classes under the supervision of their school-teacher as well as by a trained research team member.

### 3.4. Screen Time

Screen time data were obtained via the U.S. Youth Risk Behavior Surveillance Survey [29] questionnaire by self-administered questions: “How long do you watch TV per day?” and “How long do you use your computer or playing nonactive video games per day? Answers ranged from <30 m, 30 m–1 h, 1 h–1 h30, 1 h30–2 h to >2 h, being subsequently categorized from 0 to 4 (−/+). Individual scores were summed to obtain a total score for screen time, as reported in different studies [30, 31].

### 3.5. Familial Characteristics

#### 3.5.1. Parents' Occupation

Parents' occupation was categorized into ten groups (from 0 to 9) according to the Portuguese National Classification of Occupations (2010), where group 0 is the highest SES and group 9 is the lowest. Categories are as follows: (0) armed forces; (1) central administration/politicians and executive directors; (2) specialists of intellectual and scientifically activities; (3) technicians and intermediate level jobs; (4) back-office jobs; (5) security, seller, and individual services; (6) farmer and qualified workers of farm, fish, and forest; (7) industry and building qualified jobs; (8) machine and equipment operators; and (9) nonqualified jobs.

#### 3.5.2. Parental Support for Physical Activity

Participants answered a questionnaire concerning the perceived support for PA received from their parents based on the Sallis, Grossman [32] questionnaire. This validated questionnaire includes a list of items related to parental encouragement for children's PA practice [32]. The response options for all questions range from 0 (never) to 5 (very often), and the sum of the responses was computed to obtain a score for parental support.

### 3.6. Statistical Analysis

The analysis was conducted in three steps. Firstly, IBM-SPSS software version 25 was used to compute basic descriptive statistics (mean ± standard deviations). To address the first aim of the study, we ran a null model (without covariates) with three levels for each characteristic. The aim was to test for mean changes between baseline and follow-up through the analysis of *z*-test scores. To deal with aim two, a model with three levels (repeated measures (baseline and follow-up) nested within individuals, which are themselves nested within sibling pairs) was used to compute an intraclass correlation as a measure of individual tracking (ICCitrk) in all obesity markers within sib-ships. To answer aims three and four, we also relied on a multilevel model but based on two hierarchical levels, i.e., repeated measures (baseline and follow-up) nested within sibling pairs. The within and between sib-ship variances were estimated separately, and we obtained different intraclass correlations (*ρ*), with their corresponding 95% confidence intervals (%95 CI), for the three sib types for each time point (baseline and follow-up) according to an approach described by Hedeker et al. [33]. Separate models were estimated for %BF, BMI, and WC using the following set of covariates: sib types (brother-brother (BB), sister-sister (SS), and brother-sister (BS) pairs) and sib type by time point (BB as a reference in both). Also, since there were a relatively small sample of twins (*n* = 23 twin pairs of the total of 84 sibling pairs), models were further adjusted for this condition (code: 0 = no twin sib-ship; 1 = twin sib-ship) as well as an interaction between twin and time point. Finally, we also adjusted for a series of other covariates, namely, maturity offset, screen time, fruit and vegetable consumption, sugary drink consumption, physical activity, parent support for PA, and father and mother occupation. In all models, BB pairs at baseline were the reference category. When needed, covariates were centered at their respective means as generally advocated [34]. All parameters were estimated by maximum likelihood procedures [35], and the significance level was set at 5%.

## 4. Results

Descriptive statistics for the biological, behavioral, and familial characteristics are shown in Table 1. As expected, all sib types are older and more mature at follow-up compared to baseline (*p* < 0.05). Mean behavioral and familial characteristics are similar across the sib types at both baseline and follow-up (*p* < 0.05), except for BB in unhealthy diet that have lower values in follow-up compared to baseline (*p* < 0.05) and for BS in parental support for PA that reported higher support in follow-up compared to baseline (*p* < 0.05).

On average, all sib types increased their BMI and WC at follow-up compared to baseline (*p* < 0.05). SS pairs had more %BF at the follow-up compared to baseline (*p* < 0.05), but BB and BS pairs did not change significantly (*p* < 0.05). When the whole sample was considered, all subjects, on average, have higher %BF, BMI, and WC at follow-up when matched to baseline (*p* < 0.05).

Table 2 shows tracking coefficients for individual siblings within their respective sib-ships. Across all obesity markers, tracking is high, ranging from 0.90 to 0.97 (BB), 0.75 to 0.95 (SS), and 0.73 to 0.97 in (BS).

The intraclass correlation (*ρ*) values of sibling resemblance are in Table 3 and show that, regardless of the obesity marker, SS pairs resemble each other more than BB and BS pairs at both baseline and follow-up. Additionally, BS pairs were less similar in all markers at both time points. Moreover, there are slight differences in siblings' *ρ* values from baseline to follow-up in all markers, suggesting stability in siblings' resemblance across time.

Finally, Table 4 contains results showing the relevance of covariates on siblings' resemblance. The BB pairs tended to have lower values of %BF (*β* = 22.63 ± 1.91) as compared to SS pairs at both baseline (*β* = 4.06 ± 1.35) and follow-up (*β* = 5.88 ± 1.64). They also had higher WC values (*β* = 71.57 ± 2.21) when compared to SS and BS in both baseline (*β* = −4.87 ± 1.79 and *β* = −2.84 ± 1.47, for SS and BS, respectively) and follow-up (*β* = −4.58 ± 2.03 and *β* = −3.68 ± 1.55, for SS and BS, respectively). No differences were found for twins at baseline and follow-up in all obesity markers as well as in BMI in all sib types compared to BB pairs at baseline. Further, more mature subjects tended to exhibit higher values of %BF (*β* = 0.49 ± 0.23), BMI (*β* = 0.86 ± 0.12), and WC (*β* = 2.30 ± 0.27). Those who had greater screen time were also those that had, on average, more %BF (*β* = 0.27 ± 0.14), whereas those consuming more unhealthy food had lower %BF and BMI values (*β* = −0.10 ± 0.02 and *β* = −0.03 ± 0.01, for %BF and BMI, respectively). Siblings whose mothers had less qualified occupations tended to have lower BMI (*β* = −0.15 ± 0.08). Additionally, healthy diet, physical activity, parental support for PA, and father occupation did not significantly associate with siblings' %BF, BMI, and WC (*p* > 0.05).

## 5. Discussion

A high individual tracking within sib-ships was observed in all obesity markers. Previous studies with unrelated subjects that also examined tracking in obesity markers during adolescence to adulthood showed moderate-to-high values across time [36, 37]. For example, Eisenmann et al. [36] reported a moderate to relatively high tracking (expressed by autocorrelations (*r*)) in all obesity markers, namely, *r* = 0.64 in BMI, *r* = 0.44 %BF (derived from skinfolds), and *r* = 0.79 in WC from adolescence to adulthood. Moreover, Ronque et al. [38] also showed high tracking in a 3-year follow-up study (BMI = 0.94 and sum of skinfolds = 0.86) in adolescents. Altogether, these overall findings indicate that the ways these obesity markers express themselves in siblings show stability in their relative position and hence, despite their inherent plasticity during growth, track across time.

We also showed that, even with a slight variation within sib-pairs depending on the type of obesity marker, SS pairs tend to be more consistent than BB and BS pairs in their resemblance. These departures from similarity could be partially explained by sex differences in physiological, biochemical, and hormonal mechanisms during growth in overall body size and composition [39] and may clarify the absence of sameness in %BF and WC and the low BMI similarity in opposite-sex siblings. Available reports with twins [40, 41] and nuclear families [17] related to BMI described different changes depending on sex and period of life. For example, in prepubertal MZ twins (1–11 yrs), it was shown that BMI resemblance tended to increase in both boys and girls, and the same occurred to DZ boys; however, this resemblance tended to decrease in DZ girls and maintained its magnitude in opposite-sex twins [41], whereas older twins aged 16.1 to 24.5 yrs tended to stay stable in their BMI changes [40]. In the sole nuclear family longitudinal study we were able to retrieve, Hunt et al. [17] using family data with a 7-year follow-up (boys and girls mean ages at baseline were 12.4 and 11.8 yrs, respectively) also analyzed a phenotype termed Δ (changes) in three obesity markers (BMI, skinfolds, and WC) and reported lower *ρ* values than those shown in the present study. Furthermore, the authors reported within-pair *ρ* changes (BMI = 0.07, skinfolds = 0.00, and WC = 0.34) for all sib types. Moreover, Haberstick et al. [16] informed that, from adolescence to early adulthood (16–22 yrs), BB pairs tend to exhibit a similar pattern of BMI (from 0.36 (baseline) to 0.34 (follow-up)), while SS and BS pairs show a slight increase (from 0.49 to 0.55 and 0.38 to 0.43, respectively).

We claim that the discrepancy found in these results and the ones obtained in our study may be related to several factors: (i) the adjustments made for confounders, (ii) differences in study design, (iii) follow-up time, and (iv) statistical analyses. In any case, what these results tend to show is the importance of both genetic and environmental factors and probably their interactions on changes in obesity markers within the family orbit. We also contend that given the evident lack of data on genome-wide association (GWA) and gene-environment interaction studies in all markers (except for BMI), we are not yet able to present a clear picture of what is happening [42–44]. Indeed, using BMI data Felix et al. [43] GWA study signaled that the direction of the effect size for all 15 single nucleotide polymorphisms (SNPs) was identical in children and in adults. However, Graff et al. [44] showed that obesity susceptibility loci may have a comparatively stronger role during adolescence than adulthood, with variations across race/ethnic subpopulations. Additionally, using European and African American samples, Zhao et al. [42] examined social/psychosocial factors that may modify the effect of sets of genetic variants on BMI and reported that socioeconomic status (parental education) was found to modify the genetic effect in the gene/region around SNP rs9540493 on BMI only in European Americans.

Siblings' individual and familial characteristics were differently associated with obesity markers, except for biological maturation that was positively associated with all markers, suggesting that those ahead in their biological maturation have greater chances of developing obesity, and this is in line with previous findings [45, 46]. However, changes in means of obesity markers across time only differed at baseline in siblings from different types. Thus, BB pairs showed less %BF than SS pairs and higher WC than BS and SS pairs. These results indicate that despite sib-pairs (depending on sex) starting from different mean values for the obesity markers, these values tend to come closer in time. In the particular case of BMI and WC, our results confirm other studies [47, 48]. Nonetheless, available data [48, 49] related to %BF showed different tendencies: boys proportionately decrease their body fat, whereas girls increase theirs across age. Indeed, our study revealed the same tendency (see Table 3) although results were not statistically significant. A plausible reason underlying this lack of significance may be related to the fact that we only have a 2-year follow-up, and we need to expand the time window of follow-up to clarify this tendency.

Screen time only was associated with %BF, and siblings who spent more time in front of screens tended to have higher %BF. However, previous studies have shown contradictory results. For example, Chinapaw et al. [50] in a systematic review found a lack of evidence for a positive longitudinal relationship between screen time and BMI, %BF, and WC. Likewise, Fulton et al. [51] reported that even after adjusting for age and sexual maturation, screen time was unrelated to obesity markers, while Delmas et al. [52] showed positive associations in boys but not in girls. On the other hand, previous systematic reviews reported strong evidence that spending large proportions of awake time on screens was positively associated with obesity markers [53]. There are some inconsistencies between such studies, perhaps as a consequence of employing different analytical strategies along with the use of different samples and/or study designs. The direction and magnitude of the positive associations between screen time and obesity depend on the marker regardless, and our results showed that screen time strongly influenced %BF, which may contribute to the development of obesity. Additionally, in future studies, we recommend the use of different indicators of obesity in the same sample to better understand these associations. Unexpectedly and contrary to most available literature [54, 55], healthy diet was not associated with all obesity markers and unhealthy diet was negatively associated with %BF and BMI in our sample. Still, our findings were corroborated by previous studies [56–58]. For example, McNaughton et al. [58] reported no associations between BMI or waist circumference and any of the dietary patterns. On the other hand, Parnell et al. [57] reported that obese children had a significant lower intake of sugar than overweight or normal weight children. One possible explanation for these unexpected results may be related to the perception of children and adolescent with the excess of %BF and weight, which tend to underreport their real consumption. On the other hand, youth with normal weight may not be restricting their unhealthy foods compared with youth having excess of fat, which can try to reduce their weight by diminishing the consumption of these types of food.

Finally, from all familial characteristics, only mother's occupation was associated with BMI. Indeed, siblings whose mothers had less qualified occupations presented lower BMI. Socioeconomic status (SES) and its corresponding indicators (e.g., household income, parents' education, and occupation) were strongly associated with obesity markers in previous studies. However, the direction and magnitude of these associations are heterogeneous and inconsistent, ranging from negative to positive associations depending on the country and obesity marker [59]. Nevertheless, our results are similar to those presented by Gurzkowska et al. [60] in a study conducted on Polish adolescents. Although the authors had not performed any adjustment for other confounders, they found that higher SES was associated with higher weight, BMI, and WC. On the other hand, a study conducted by Costa de Oliveira Forkert et al. [61] in European (Germany, Sweden, Greece, Italy, Spain, Hungary, Belgium, France, and Austria) and Brazilian adolescents found different results for WC. Here, both parental education and father's occupation levels were negatively associated with WC in European girls, while in boys only mother's occupation level was inversely associated with WC. However, among Brazilian adolescents, no significant associations were found. Notwithstanding, this study only adjusted its analysis for age and focused on European versus Brazilian, which could explain the different directions of associations compared to our study. Altogether, these results suggest that SES is an important factor that influences not only individual development, at large, but also its obesity development. However, it is noteworthy to mention that the direction of SES influence is not consistently universal, and programs for preventing obesity should be defined and tailored according to SES specificities.

Notwithstanding the relevance of our results, this study has some limitations. For example, (i) participants were not recruited from all Portuguese regions, which restricts the generalization of the results to the whole Portuguese population; however, this issue is common in family and twin studies. (ii) The use of questionnaires to obtain information about physical activity, screen time, and diet is prone to errors, although the questionnaires have been applied in controlled conditions. Further, these questionnaires are frequently used, and previous studies have confirmed their reliability. (iii) It may be possible that the variance components, as well as intraclass correlation estimates, could be different if we had precise information about twin zygosity. Yet, in results not shown, we tested different models with and without twin data and no substantive differences were found suggesting that such absence did not compromise our results.

## 6. Conclusions

In overall terms, our findings report increases in BMI and WC for all sib types for 2 years of follow-up, as well as for %BF in SS pairs. Obesity markers tend to track across time within sib-ships, whereas sibling resemblance tends to be consistent over time. Additionally, our data reinforce the idea that both individual and familial characteristics may exert divergent influences on each obesity marker development. Hence, we suggest that prevention strategies need to consider the complex and intertwined relationships of both familial and biological characteristics that may provide new insights in obesity control. Therefore, when developing suitable interventions aiming to prevent obesity, it is crucial to consider a target marker, fashioning different programs for distinct groups based on their individual and familial characteristics. Finally, we need more longitudinal studies based on larger family-based data relying on a multilevel approach combined with appropriate statistical models. We are convinced that this endeavor will help to build a more comprehensive understanding of this complex interaction between nature and nurture in obesity phenomena.

## Figures and Tables

**Table 1:** Descriptive statistics (means and standard deviations (SD) and frequencies (%)) for all sib types at baseline and follow-up.

	Brother-brother (*n* = 50)		*z*	Brother-sister (*n* = 84)		*z*	Sister-sister (*n* = 34)		*t*	All siblings (*n* = 168)		*z*
	Baseline	Follow-up		Baseline	Follow-up		Baseline	Follow-up		Baseline	Follow-up	
	Mean ± SD	Mean ± SD		Mean ± SD	Mean ± SD		Mean ± SD	Mean ± SD		Mean ± SD	Mean ± SD	
*Individual characteristics*												
Chronological age (yr)	12.3 ± 1.4	14.6 ± 1.6	19.98^*∗∗∗*^	12.2 ± 1.4	14.3 ± 1.3	14.02^*∗∗∗*^	11.8 ± 1.3	14.2 ± 1.3	17.10^*∗∗∗*^	12.2 ± 1.4	14.4 ± 1.4	25.48^*∗∗∗*^
Biological maturation (yr)	−0.9 ± 1.5	0.8 ± 1.7	16.72^*∗∗∗*^	−0.9 ± 1.2	0.6 ± 1.2	13.64^*∗∗∗*^	-0.8 ± 1.1	0.8 ± 0.9	14.51^*∗∗∗*^	-0.9 ± 1.3	0.6 ± 1.3	23.60^*∗∗∗*^
Body fat (%)	19.4 ± 5.9	19.4 ± 6.0	−0.06^ns^	21.8 ± 6.0	22.3 ± 5.9	1.21^ns^	25.1 ± 4.4	27.8 ± 5.3	6.11^*∗∗∗*^	21.8 ± 5.9	22.5 ± 6.5	3.14^*∗∗*^
Body mass index (kg/m^2^)	19.7 ± 3.5	20.6 ± 3.7	6.54^*∗∗∗*^	18.9 ± 2.6	19.7 ± 2.6	3.94^*∗∗∗*^	19.2 ± 3.1	20.7 ± 3.4	7.13^*∗∗∗*^	19.2 ± 3.0	20.2 ± 3.1	8.34^*∗∗∗*^
Waist circumference (cm)	67.3 ± 9.2	70.2 ± 10.2	8.15^*∗∗∗*^	64.4 ± 5.7	66.1 ± 5.5	4.50^*∗∗∗*^	63.1 ± 6.6	65.8 ± 6.8	5.49^*∗∗∗*^	65.0 ± 7.2	67.3 ± 7.7	9.51^*∗∗∗*^

*Behavioral characteristics*												
Screen time	3.4 ± 2.6	3.6 ± 2.2	0.63^ns^	3.0 ± 2.2	2.9 ± 1.9	−0.26^ns^	2.8 ± 2.2	2.4 ± 2.1	−1.00^ns^	3.1 ± 2.3	3.0 ± 2.1	−0.22^ns^
Total physical activity	8.2 ± 2.0	8.5 ± 1.6	1.09^ns^	7.6 ± 2.2	8.0 ± 1.4	1.62^ns^	7.8 ± 1.3	7.7 ± 1.9	−0.30^ns^	7.8 ± 2.0	8.1 ± 1.6	1.73^ns^
Healthy diet	44.0 ± 18.3	42.5 ± 18.7	−0.48^ns^	42.1 ± 5.2	39.6 ± 13.5	−1.47^ns^	41.0 ± 13.9	38.4 ± 20.4	−1.02^ns^	42.5 ± 15.9	40.2 ± 16.7	−1.62^ns^
Unhealthy diet	29.1 ± 19.3	24.4 ± 15.5	−2.08^*∗*^	22.6 ± 14.1	21.2 ± 14.2	−1.13^ns^	15.0 ± 7.4	13.5 ± 7.5	−1.34^ns^	23.0 ± 15.6	20.6 ± 14.0	23.03^*∗*^

*Familial characteristics*												
Father occupation	6.0 ± 2.7	6.0 ± 2.7	−0.00^ns^	5.6 ± 2.5	5.6 ± 2.5	1.01^ns^	6.0 ± 2.0	6.1 ± 2.1	1.79^ns^	5.8 ± 2.5	5.9 ± 2.5	1.98^ns^
Mother occupation	6.7 ± 2.2	7.1 ± 2.1	0.86^ns^	5.6 ± 2.7	5.6 ± 2.7	−1.75^ns^	6.2 ± 2.8	6.2 ± 2.8	0.00^ns^	6.2 ± 2.6	6.2 ± 2.6	0.27^ns^
Parental support for PA	11.8 ± 8.1	11.3 ± 7.3	−0.42^ns^	10.5 ± 6.5	11.9 ± 6.0	2.00^*∗*^	11.2 ± 6.1	12.7 ± 5.9	1.72^ns^	11.0 ± 6.9	11.9 ± 6.4	1.77^ns^

^*∗*^ *p* < 0.05; ^*∗∗*^*p* < 0.01; ^*∗∗∗*^*p* < 0.001; ns = nonsignificant.

**Table 2:** Individual siblings tracking (ICC_itrk_) coefficients and 95% CI within sib-ships for the obesity markers with twins and without twins data.

Tracking coefficients	%BF ICC_itrk_ (95% CI)	WC ICC_itrk_ (95% CI)	BMI ICC_itrk_ (95% CI)
Brother-brother	0.90 (0.83–0.95)	0.96 (0.92–0.98)	0.97 (0.94–0.98)
Sister-sister	0.91 (0.81–0.96)	0.97 (0.93–0.99)	0.75 (0.57–0.87)
Brother-sister	0.97 (0.94–0.98)	0.93 (0.87–0.97)	0.73 (0.55–0.85)

**Table 3:** Intraclass correlation coefficients (*ρ*) and 95% CI within sib-ships for the obesity markers with twins and with twins data.

Tracking coefficients		%BF	WC	BMI
Brother-brother	Baseline	0.45 (0.19–0.75)	0.37 (0.12–0.72)	0.24 (0.04–0.71)
	Follow-up	0.39 (0.13–0.73)	0.36 (0.11–0.72)	0.23 (0.03–0.72)
Sister-sister	Baseline	0.55 (0.21–0.84)	0.53 (0.21–0.83)	0.62 (0.31–0.86)
	Follow-up	0.59 (0.27–0.85)	0.47 (0.15–0.81)	0.63 (0.31–0.86)
Brother-sister	Baseline	0.03 (0.00–1.00)	0.01 (0.00–1.00)	0.07 (0.00–0.88)
	Follow-up	0	0	0.19 (0.03–0.62)

**Table 4:** Parameter estimates and variance components for each obesity marker.

Fixed effects	%BF	BMI	WC
	Estimate ± SE	Estimate ± SE	Estimate ± SE
Intercept (BB baseline)	22.63 ± 1.91^*∗∗∗*^	22.09 ± 0.95^*∗∗∗*^	71.57 ± 2.21^*∗∗∗*^
BB follow-up	−1.42 ± 1.55	−0.38 ± 0.80	−0.02 ± 2.09
SS baseline	4.06 ± 1.35^*∗∗*^	−1.04 ± 0.80	−4.87 ± 1.79^*∗∗*^
SS follow-up	5.88 ± 1.64^*∗∗∗*^	−0.70 ± 0.95	−4.58 ± 2.03^*∗*^
BS baseline	2.11 ± 1.25	−1.00 ± 0.59	−2.84 ± 1.47^*∗*^
BS follow-up	1.96 ± 1.33	−1.22 ± 0.65	−3.68 ± 1.55^*∗*^
Twins baseline	1.80 ± 1.05	0.39 ± 0.55	1.11 ± 1.21
Twins follow-up	0.72 ± 1.47	0.28 ± 0.78	−0.15 ± 1.65
Maturity offset	0.49 ± 0.23^*∗*^	0.86 ± 0.12^*∗∗∗*^	2.30 ± 0.27^*∗∗∗*^
Screen time	0.27 ± 0.14^*∗*^	0.10 ± 0.07	0.14 ± 0.16
Healthy diet	0.01 ± 0.02	0.00 ± 0.01	-0.02 ± 0.02
Unhealthy diet	−0.10 ± 0.02^*∗∗∗*^	−0.03 ± 0.01^*∗*^	-0.01 ± 0.02
Physical activity	−0.22 ± 0.15	−0.04 ± 0.10	−0.09 ± 0.22
Parental support for PA	−0.05 ± 0.05	−0.03 ± 0.03	−0.07 ± 0.06
Father occupation	−0.21 ± 0.15	0.02 ± 0.02	−0.03 ± 0.17
Mother occupation	−0.01 ± 0.14	−0.15 ± 0.08^*∗*^	−0.07 ± 0.06

*Variance components (σ* ^*2*^)			
Between siblings'			
BB baseline	16.82 ± 8.42	2.47 ± 2.14	22.82 ± 13.47
SS baseline	7.52 ± 4.35	4.63 ± 2.24	16.24 ± 8.85
BS baseline	0.87 ± 4.80	0.43 ± 0.92	0.32 ± 4.37
BB follow-up	12.59 ± 7.21	2.46 ± 2.27	27.94 ± 16.78
SS follow-up	11.57 ± 6.07	6.00 ± 2.93	16.59 ± 10.37
BS follow-up	0	1.11 ± 0.93	0
Within siblings'			
BB baseline	20.20 ± 6.02	7.69 ± 2.23	39.22 ± 11.43
SS baseline	6.23 ± 2.33	2.80 ± 0.99	14.26 ± 5.04
BS baseline	29.67 ± 6.70	5.29 ± 1.20	27.04 ± 6.10
BB follow-up	19.83 ± 5.81	8.41 ± 2.39	49.66 ± 14.14
SS follow-up	8.04 ± 2.91	3.54 ± 1.32	18.94 ± 6.90
BS follow-up	32.20 ± 5.00	4.71 ± 1.04	25.19 ± 3.94
Log likelihood	−1026.99	−803.19	−1073.55

^*∗*^ *p* < 0.05; ^*∗∗*^*p* < 0.01; ^*∗∗∗*^*p* < 0.001.

## Data Availability

The Portuguese sibling data used to support the findings of this study are available from the corresponding author upon request.
